# Structural Requirements of N-Substituted Spiropiperidine Analogues as Agonists of Nociceptin/Orphanin FQ Receptor

**DOI:** 10.3390/ijms12128961

**Published:** 2011-12-06

**Authors:** Pingping Bao, Xiaole Zhang, Hong Ren, Yan Li, Zulin Mu, Shuwei Zhang, Guohui Li, Ling Yang

**Affiliations:** 1 Department of Materials Science and Chemical Engineering, Dalian University of Technology, Dalian, Liaoning 116023, China; E-Mails: hedybao@dlut.edu.cn (P.B.); muzulin7@gmail.com (Z.M.); zswei@dlut.edu.cn (S.Z.); 2 Department of Mathematical Sciences, Dalian University of Technology, Dalian, Liaoning 116023, China; E-Mail: zxlfree@foxmail.com; 3 Department of Ophthalmology, Qi Lu Hospital, Medical School of Shandong University, Jinan 250012, China; E-Mail: renhong999@sina.com; 4 Laboratory of Molecular Modeling and Design, State Key Laboratory of Molecular Reaction Dynamics, Dalian Institute of Chemical Physics, Chinese Academy of Sciences, Dalian 116023, China; E-Mail: ghli@dicp.ac.cn; 5 Lab of Pharmaceutical Resource Discovery, Dalian Institute of Chemical Physics, Graduate School of the Chinese Academy of Sciences, Dalian, Liaoning 116023, China; E-Mail: yling@dicp.ac.cn

**Keywords:** NOP agonist, N-substituted spiropiperidine analogues, 3D-QSAR, molecular docking, molecular dynamics

## Abstract

The nociceptin/orphanin FQ (NOP) receptor is involved in a wide range of biological functions, including pain, anxiety, depression and drug abuse. Especially, its agonists have great potential to be developed into anxiolytics. In this work, both the ligand- and receptor-based three-dimensional quantitative structure–activity relationship (3D-QSAR) studies were carried out using comparative molecular field analysis (CoMFA) and comparative molecular similarity indices analysis (CoMSIA) techniques on 103 N-substituted spiropiperidine analogues as NOP agonists. The resultant optimal ligand-based CoMSIA model exhibited *Q^2^* of 0.501, *R^2^*_ncv_ of 0.912 and its predictive ability was validated by using an independent test set of 26 compounds which gave *R^2^*_pred_ value of 0.818. In addition, docking analysis and molecular dynamics simulation (MD) were also applied to elucidate the probable binding modes of these agonists. Interpretation of the 3D contour maps, in the context of the topology of the active site of NOP, provided insight into the NOP-agonist interactions. The information obtained from this work can be used to accurately predict the binding affinity of related agonists and also facilitate the future rational design of novel agonists with improved activity.

## 1. Introduction

NOP, the nociceptin/orphanin peptide, is a 17-amino acid neuropeptide which was discovered in 1995 [1,2]. Though structurally related to the opioid peptidedynorphin A [3,4], NOP lacks the *N*-terminal tyrosine necessary for activation of μ-, κ- and δ-opioid receptors and therefore does not bind to the opioid receptors. Actually, as an endogenous ligand, it binds only to its own receptor, *i.e.*, the NOP receptor (also known as ORL1, OP4 or LC132) which was cloned in 1994 [5] and named after this ligand. NOP receptor belongs to the transmembrane G-protein coupled receptor family, and is widely distributed in the central nervous system with the highest density in the forebrain, brainstem, dorsal and ventral horns of the spinal cord. Besides, it is also present in the peripheral nervous system as well as in some non-neural tissues (epidermis, immunocytes, and vascular endothelium) [6–8]. Due to the therapeutic potential of the NOP receptor, it has received considerable attention in research since it was cloned.

The agonists of NOP receptor have a broad therapeutic potential [9] to be used as antitussives, anxiolytics, vasodilators, hypotensives and in the treatment of neuropathic pain, drug dependence, urinary incontinence, congestive heart failure, and anorexia [10]. Thus scientists have spared no effort in development of NOP agonists, ending up with a variety of reported agonists. Generally, they can be divided into two classes: the peptide ligand and non-peptide ligand. For the first class of peptide ligand, the most typical one is the NOP which not only binds to, but also activates, the normal function of NOP receptor. In addition, some nociceptin-related peptides were also reported with high NOP binding affinities as NOP agonists [11–18]. As to the second class of non-peptide agonists, several groups of NOP ligands based on structural differences have been discovered, including piperidines, nortropanes, spiropiperidines, 4-amino-quinolines and quinazolines, and others [10]. Among these, the most extensively studied is the triazaspirodecanone Ro 64-6198 synthesized by Roche group [19], which has indeed become a valuable pharmacological tool in determining the potential of the NOP receptor as a therapeutic target. Based on this triazaspirodecanone, a series of spiropiperidines were further synthesized by optimization of a high-throughput screening lead containing the triazaspirodecanone (comprising the A and B moieties of the proposed pharmacophore) and a substituted 2-tetralinyl moiety as the lipophilic C moiety directly linked to the basic piperidine nitrogen [20]. Intracerebral infusions of NOP or systemic injections of the NOP receptor agonist, Ro64-6198, were found to affect neuroendocrine function, feeding, locomotion, learning and memory, anxiety, stress response and sexual behavior [21–28]. Due to the therapeutical potential of spiropiperidines, a series of N-substituted analogs based on the spiropiperidine analogues were synthesized by Caldwell JP, which exhibited high binding affinity to the NOP receptor [29,30].

The comparative molecular field analysis (CoMFA) method proposed in 1988 by Crammer *et al.* and the subsequently developed comparative molecular similarity indices analysis (CoMSIA) method have been extensively used in many present practices of drug discovery and development as three-dimensional quantitative structure-activity relationship (3D-QSAR) approaches [31–37], due to their outstanding advantages of time-saving, cost-reducing as well as the highly efficient *in silico* screening and prediction of candidate drugs. Until now, *in silico* studies on spiropiperidine analogues as agonists of NOP receptors are still very limited, especially 3D-QSAR studies. Therefore, a 3D-QSAR analysis on this kind of NOP ligands should be of great significance.

In the present work, a total of 103 N-substituted spiropiperidine analogues were computationally studied to build 3D-QSAR models using CoMFA and CoMSIA methodologies [38]. The predictive abilities of the obtained models were validated statistically by an independent test set of compounds. Furthermore, a combined *in silico* approach including docking analysis, and molecular dynamics (MD) simulation was also employed to elucidate the probable binding modes of these agonists at the active site of the NOP receptor. We hope this study will support the use of spiropiperidine analogues as a potential therapeutic agent by targeting NOP and be helpful in designing novel and more effective NOP agonists as desired.

## 2. Results and Discussion

### 2.1. CoMFA and CoMSIA Statistical Results

Since the alignment of compound structures plays an important role in developing successful 3D-QSAR models [39], two rules (both ligand-based and docking-based) were adopted to align the dataset to derive reliable models. The results obtained from both models using the same training set of 81 compounds are summarized in Table 1. A number of statistical parameters, *i.e.*, the *Q^2^*, non-cross-validated correlation coefficient (*R^2^*_ncv_), SEE, and F-statistic values, are analyzed to evaluate the quality of the models.

In both CoMFA and CoMSIA analyses, ligand-based alignment modeling leads to models with larger *R*^2^_cv_, *R*^2^_ncv_, *R*^2^_pred_ values than the corresponding models obtained by the receptor-based alignment modeling. Therefore, we mainly focussed on the ligand-based 3D-QSAR models for further analysis. In addition, since the five (steric, electrostatic, hydrophobic, and H-bond donor and acceptor) field descriptors may not be completely independent of each other and such dependency among individual fields may reduce the model significance and generalization [40,41], all possible combinations of the descriptors were used to derive models for avoiding the risk of omitting possible optimal model and to explore the best combination use of the descriptors for model generation. Finally, a CoMSIA model established by using the steric, electrostatic, hydrophobic and hydrogen bond donor field descriptors appears to be superior to all other models derived, whose statistical results are listed in Table 1. Using seven PLS components, this model yields statistical results of *Q*^2^ = 0.501, *R*^2^_ncv_ = 0.912, SEE = 0.250 and *F* = 108.309 with steric (12.4%), electrostatic (38.7%), hydrophobic (24.4%) and H-bond donor (24.5%) field contributions, proving its correct internal predictive capability.

**Figure 1:**
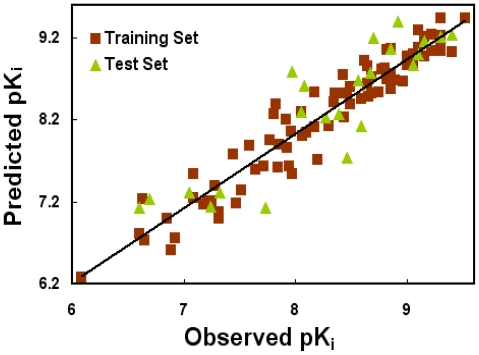
The correlation plots of predicted *versus* actual pK_i_ values using the training (filled red squares) and test (filled green triangles) sets based on the optimal CoMSIA model. The solid lines are the regression lines for the fitted and predicted bioactivities of training and test compounds, respectively.

Generally speaking, a *Q*^2^ > 0.5 is considered proof of acceptable internal predictive ability [42]. What’s more, the high *R*^2^_ncv_ and *F* values along with the low SEE values should also be considered as the foundation of a reliable QSAR model [43]. However, due to chance correlation or structural redundancy, sometimes it is found that some models derived from the training set molecules with randomized activity possess high *Q*^2^ values, but show unfavorable predictivity for prediction of unknown molecules [33,44]. Hence, the extensively accepted leave-one-out (LOO) cross-validated *Q*^2^ is insufficient to assess the predictive power of the QSAR models [45]. In light of such risks, we validated the models by predicting the activity of an external test set composed of 22 NOP agonists. As a result, a predictive coefficient *R*^2^_pred_ of 0.818 was achieved verifying the good external predictive efficacy of the model (Table 1). Figure 1 illustrates the correlation plot of experimental *versus* predicted pKi values of the training (filled red square) and test (filled green triangle) sets based on the optimal CoMSIA model. Clearly, a good correlationship is observed from this figure since the predicted values are almost as accurate as the experimental activities for the whole dataset, and all points are rather uniformly distributed around the regression line, indicating no existence of systematic errors in the method. This good agreement between the predicted and experimental activity data proves the satisfactory predictive ability of the CoMSIA model.

### 2.2. 3D-QSAR Contour Maps

**Figure 2:**
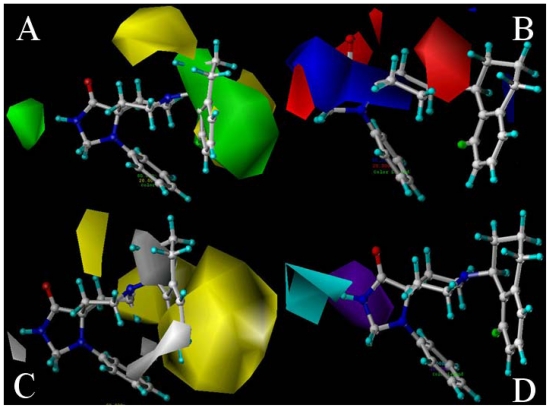
CoMSIA stdev*coeff contour plots for NOP in combination with compound **32**. (**A**) Steric (green/yellow) contour map. Green contours indicate regions where bulky groups increase activity; yellow contours indicate regions where bulky groups decrease activity; (**B**) Electrostatic contour map (blue/red). Blue contours indicate regions where positive charges increase activity; red contours indicate regions where negative charges increase activity; (**C**) Hydrophobic contour map (yellow/white). Yellow contours indicate regions where hydrophobic substituents enhance activity; white contours indicate regions where hydrophobic substituents decrease activity; (**D**) CoMSIA contour maps illustrating hydrogen-bond donor features. The cyan contour represents the H-bond donor favored region, purple indicates the disfavored region.

The 3D-coefficient contour plots are beneficial to identify important regions where some changes in the interaction fields can affect the biological activity, and may also be of help to identify the possible interaction sites of the biochemical system. Thus presently, the optimal ligand-based CoMSIA model is selected for each conformation to construct the stdev*coeff contour maps to view the field effects on the target features due to its good internal and external predictive powers. The maps generated depict regions having scaled coefficients greater than 80% (favored) or less than 20% (disfavored). To aid in visualization, the most active compound 32 is shown as template molecule with the contour maps (Figure 2).

The CoMSIA steric contour plot for the most active compound **32** is displayed in Figure 2A, where the sterically favored regions are shown in green and disfavored regions in yellow, respectively. As seen from this picture, some green contours are mapped near position-1 of ring A, positions-11 and -12 of ring D and positions-19 and -20 of ring E, suggesting that bulkier groups are favored at these positions. The green contour around position-1 is well consistent with the higher potency of compound **53** with a bulkier substituent (CH_3_OC(O)CH_2_-) (p*K*_i_ = 7.71) than compound **15** without substituent (p*K*_i_ = 7.63) at position-1 of ring A. The higher potency of compound **38** (p*K*_i_ = 8.85, with a CH_3_ group) than **36** (p*K*_i_ = 7.96, without substituent) is also such a case. A few residues located around positions-11, -12, -19 and -20 lead to a large empty space at these positions (as shown in Section 2.3), which can interpret the presence of green contours at these positions. Two yellow contour maps appeared above ring C and between ring C and ring D, respectively, implying that bulkier substituents at these positions may decrease the activity; the reason may be that there is no such bulky substituent at these positions of both rings C and D in all molecules of the dataset.

Figure 2B depicts the electrostatic contour maps obtained from the CoMSIA model, where blue contours represent the favorable electropositive regions and red contours account for the favorable electronegative regions, respectively. A large blue contour extending from position-1 to position-4 indicates that electropositive groups are preferred here. Compound **62** with substituent of (CH_3_)_2_N(CH_2_)_2_- at position-1 shows higher activity than compound **56** with substituent of NH_2_(CH_2_)_2_- due to the stronger electronegativity of the former substituent. Besides the large blue contour, a red contour can be seen near position-1 of ring A indicating that this position is sensitive to electrostatic substituents. This phenomenon may have something to do with the atom N (electronegative atom) of ligand 32 at position-1. In addition, the atom O (electronegative atom) of ligand 32 at position-5, may be the reason for the small red contour showing around here. The function and location (next to position-1) of atom O at position-5 may lead to the red contour appearing around position-1. Also a blue contour located at positions-11 of ring D, suggests the possible help of electropositive substituents, improving the binding affinity. Actually, this may be due to the existence of the electro-negative amino acids Asp130 and Asp209 in the binding pocket, as discussed later in Section 2.3, because of the lack of any substituents in these areas. In addition, a large red contour map is seen around position-8, which is caused by the electronegative atom N and an electro-positive amino acid Arg302 (as discussed later in Section 2.3).

**Figure 3:**
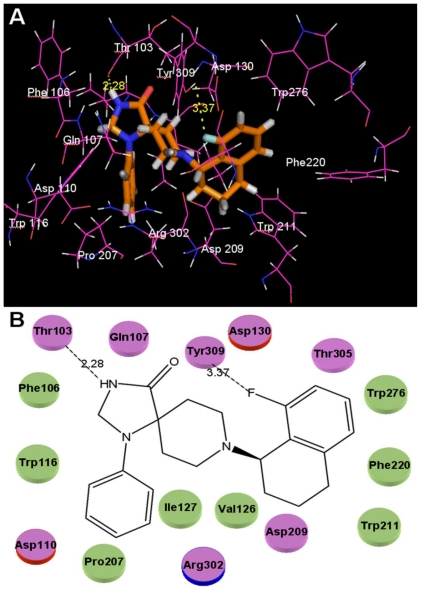
The binding site formed around compound **32**. (**A**) Interactions with the key amino acids in the binding pocket. The dashed lines show the formation and distance (in Å) of the hydrogen bonds. Active site amino acid residues are represented as lines, the agonist is shown as stick model, respectively; (**B**) The pink cylinders represent the polar residues, where, especially the ones with red circles, represent the acid residues and the one with a blue circle represents basic residue. The green cylinders represent non-polar residues. Dash lines represent the H-bond interactions.

Figure 2C shows the CoMSIA hydrophobic contour map, where the yellow (hydrophobic favorable) and white (hydrophobic unfavorable) contours represent 80% and 20% contributions respectively. Substitutions by hydrophobic groups like -Cl and -F at positions-18 and -19 are extended to the yellow contours resulting in a higher NOP activity, which can be illustrated by the example that compound **33** with -F at position-18, and compound **32** with -F at position-19 all exhibit higher activities than compound **29** without any substituent at either positions-18 or -19, respectively. The existence of atom F at position-19 may be the reason for the yellow contour extending. Furthermore, two large yellow regions are observed above positions-6 and -7 and around position-9 of ring C, respectively. Also some white regions are observed close to position-1 of ring A, ring B, positions-11 and -12 of ring C and position-17 of ring E indicating that hydrophilic groups here are helpful for the activity. The fact that compound **83** with hydrophilic group -(CH_2_)_2_OH has higher potency than compound **103** with hydrophobic group -NHBu at position-1 verifies this conclusion. What’s more, compound **54** with hydrophilic substituent-(CH_2_)_2_OH at position-1 shows higher binding affinity than both compounds **51** (with hydrophobic group -Bu) and **52** (with hydrophobic group *i*-Amyl-). The white contour around positions-11 and -12 of ring C and position-17 of ring E may be attributed to the existence of the hydrophilic residues (Asp110, Asp209, Asp130, Thr103, Thr305, Tyr309, Gln107 and Arg302) in binding pocket, as discussed later in Section 2.3 and shown in Figure 3B.

In Figure 2D, the CoMSIA hydrogen-bond donor plot, the cyan contours indicate regions where hydrogen bond donor substituents on the ligands are favored and the purple contours represent areas where hydrogen bond donor substituents on agonists are disfavored. As seen from the picture, a large cyan contour appears around position-1. Its appearance was due to the atom N at position-1 acting as an H-bond donor and interacts with the key amino residues around the position (as discussed later in Section 2.3). The only structural difference of compounds **72**~**79**, lies in the substituent at position-1 (such as the EtNHCH_2_CH_2_- and BuNHCH_2_CH_2_- groups) which can serve as the H-bond donor for interaction with the surrounding environment. Thus, they all exhibit higher p*K*_i_ values than compound **80** with H-bond accepter substituent (

**Figure:**
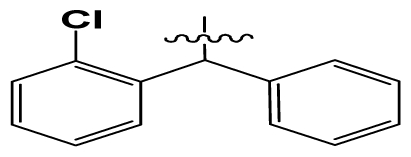


) at position-1, providing powerful proof for the conclusion. The atom N and atom O located at position-1 and position-5 respectively, both of which can act as H-bond donors, may be the reason for the large purple area around these positions.

### 2.3. Docking Studies

Due to the important role in the rational design of a drug, docking is often used to find the optimal orientation of a ligand in the binding to its pharmaceutical target [46]. In our work, the whole dataset of 103 compounds were docked into the possible active site of NOP receptor crystal structures, and the optimal conformations of these compounds were determined. The results show that all molecules in the series were well placed in the active site demonstrating the rationality and reliability of the docking model. The binding mode of the highly potent compound **32** docked into the receptors is taken as an example and shown in Figure 3. As observed from this figure, the ligand core is anchored in the binding site via hydrophobic interactions and two H-bonds are identified as potential factors influencing the high binding affinity of compound **32**. The specific binding interactions are analyzed in detail as follows.

As seen from the picture, compound **32** is actually docked into a basically hydrophobic pocket which is formed by Trp116, Trp211, Trp276, Val126, Pro207, Phe106, Phe220, Ile127, and Phe106. This finding reaches good agreement with the previous CoMSIA hydrophobic contour maps analysis in Figure 2C, that most hydrophobic residues around the binding ligand are consistent with those yellow contours appearing around position-9 of ring C and positions-18 and -19 of ring E. In addition, the presence of several hydrophilic residues (Asp209, Asp130, Thr103, Thr305, Tyr309 and Arg302) at positions-1, -11, -12 and -17 also conforms well with the white contours in Figure 2C.

In addition, altogether two H-bonds are observed in Figure 3 playing crucial roles in anchoring the ligand in the binding site. The -NH- of ring A as the hydrogen bond donor forms an H-bond with Thr103 with a distance of 2.28 Å. This observation correlates well with the cyan contour located around position-1 (representing the H-bond favored region) in previous CoMSIA hydrogen bond donor contour maps (Figure 2D). Besides this, another H-bond is also observed in the docking pocket, the one between the F atom at position-19 and Tyr309 (3.37 Å), which acts as a supplement for the contour map.

Our docked model also shows a comparatively large empty space around positions-11 and -12 of ring D and positions-19 and -20 of ring E, indicating that in these regions the steric interaction may be favorable. The conclusions are similar to the previous CoMSIA contour maps in Figure 2A that large blue contours show around positions-11 and -12 of ring D and positions-19 and -20 of ring E, respectively. From Figure 3A, we can see many key amino acid residues around rings C and D, which correlates well with the two yellow contours as shown in Figure 2A. Quite a lot of residues appear around position-1 creating no empty space, in contrast to the result obtained from the analysis of CoMSIA steric contour maps. The fact that position-1 plays a key role in CoMSIA field analysis (discussed in Section 2.2) may account for the difference, which in turn leads to its interaction with many residues (Trp116, Thr103, Val126 and Phe106). Several residues are also observed around rings B and C, giving supplement for the steric interactions around them, which indicates that bulky substituents around rings B and C should be harmful to the increase of binding affinity.

All in all, the docking results and the 3D contour maps complement and validate each other, indicating that the QSAR models generated in the present study are reasonable and could be used to derive useful information in the future rational design of NOP agonists.

### 2.4. Molecular Dynamics Simulations

Since our molecular docking process does not take the protein flexibility into consideration, presently 5 ns molecular dynamics simulations of NOP receptor with ligand 32 were carried out on the basis of the docked complex structure for the purpose of “drawing” a dynamic picture of the conformational changes in the NOP receptor binding site.

**Figure 4:**
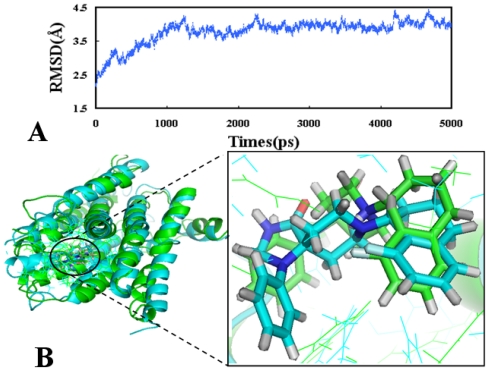
MD simulation results: (**A**) Plot of the root-mean-square deviation RMSD of docked complex *versus* the MD simulation time in the MD-simulated structures; (**B**) Structural superposition of the MD simulation and the initial structure for NOP receptor. The projection highlights the superimposed backbone atoms of the average structure of the last 1 ns of the MD simulation (green) and the initial structure (cyan) for compound 32 with NOP complex.

The RMSDs (root-mean-square deviation) of the trajectory in regard to the initial structure ranging from 2.5 to 4.5 Å are presented in Figure 4A. As a result, after 2500 ps the RMSD of the complex attains about 4.0 Å and almost remains this value for the whole process. This clearly indicates 20 metastable conformations after 2500 ps of simulation for the docked complex structure. Figure 4B depicts a superposition of the average structure for the last 1 ns and the docked structure. The right hand side picture of Figure 4B is an enlarged view of the superposition of an average structure for the last 1 ns, and the docked structure of ligand 32, where the cyan stick represents the initial structure of the docked complex and the green stick represents the MD-simulated structure, respectively. Ligand 32 is shown in blue for the initial complex and, separately, in green for the final average complex. Obviously, there are no significant changes in both the protein and ligand 32 superpositions between the docked structure and the average structure obtained from MD simulations, which verifies the reasonability of the docking model.

## 3. Discussion

The development of nociceptin/orphain FQ receptor agonists has been a hot topic in research fields for a long time. However, until now the *in silico* study on N-substituted spiropiperidine-based NOP agonists is seldomly reported except for the work of Luo HB *et al.* in 2010, where a dataset composed of 67 spiropiperidine analogues was investigated using the CoMFA approach [47]. By comparing the results of their work with ours, both similarities and differences exist, including the quantitative change of the number of molecules used in the dataset (67 in theirs, which is actually a subset of our dataset composed of 103 NOP agonists).

**Figure 5:**
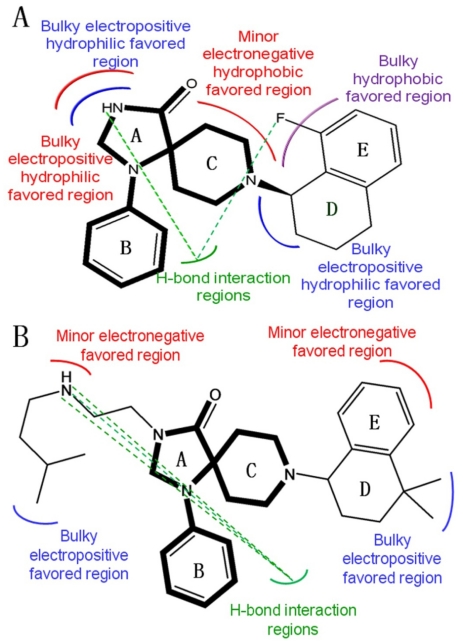
Comparison of the interaction features of (**A**) ligand 32 and (**B**) compound **P67** [47] with NOP receptor.

In detail, both theirs and our skeletons of the molecules consist of heterocyclic ring A with variations of substituents at position-1, benzene ring B and heterocyclic ring C with variations of substituents at position-3. In activity (p*K*_i_), their activity ranges from 0.4 to 234 nM while ours varies from 0.3 to 824 nM, respectively. Although both theirs and our work adopts the molecular docking method, they only docked their highly potent molecular P67 while we docked the whole dataset of 103 compounds into the binding pocket of the target. Furthermore, based on the molecular dynamics analysis we carried out, the interaction features of these spiropiperidine analogues with the NOP receptor were further investigated and validated. Figure 5 summarizes the binding modes of each work, where the similarities and differences of the two papers are easily observed.

Figure 5A displays the key structural features impacting the activity obtained from our present work and Figure 5B displays the features obtained by Luo *et al.*, respectively. The most active NOP agonist in each respective dataset, *i.e.*, ligand 32 in ours and compound **P67** in Luo’s dataset is shown as a template in Figure 5A and B respectively. In Figure 5, green dash lines represent H-bond interaction regions and other curved lines represent the specific interaction regions.

As seen from the figure, the two works draw two similar conclusions that: (i) areas around position-1 of ring A are electrostatic-sensitive, which have both electropositive and electronegative favored regions; (ii) Area around ring D is the bulky electropositive favored region.

Despite the above similarity, the difference in structural features of the two works is also distinct: (i) the interactions and main amino acid residues around the atom N of ring A are different. The reason might be that compound **P67** (in Figure 5B) has a substituent of an electropositive group (*i*-AmylNH(CH_2_)_2_-) at the position, which affects the interactions between compound **P67** and relevant residues greatly; (ii) A minor electronegative hydrophobic favored region is observed around ring C for compound **32** while it does not exist for compound **P67**. The reason is that our results are based on the optimal CoMSIA model derived by using of steric, electrostatic, hydrophobic and hydrogen-bond donor field descriptors while Luo’s results were obtained from a CoMFA model with only the steric and electrostatic field descriptors employed; (iii) The size of the binding spaces for the two datasets are different, due to the fact that compound **32** is surrounded by more amino residues (Asp130, Thr305, Ile127 and Pro207) than compound **P67** [48,49]; (iv) The interactions around ring E are different for the two compounds. Compound **P67** has a minor electronegative favored region around ring E while compound **32** has a bulky hydrophobic favored region around ring E, which may be due to the fact that compound **P67** has two polar residues Asp290 and Tyr 210 surrounding this region in the pocket, while compound **32** is surrounded by some non-polar residues such as Phe220, Trp211 and Trp276; (v) As for the H-bonds, ligand 32 forms two H-bonds, while compound **P67** produces three. The two H-bonds of ligand 32 are explained in Section 2.3. The three H-bonds of compound **P67** are formed by atom N at the substituent of ring A with related key amino residues, one is formed with Asp110 and the other two are formed with Arg302. The difference in both the molecular structure and the surrounding amino residues of ligands 32 and P67 leads to the different H-bond interactions. All in all, these conclusions would help guide the further development of N-substituted spiropiperidine-based NOP agonists with improved potency.

## 4. Materials and Methods

### 4.1. Database and Biological Activity

Discarding those compounds with unspecified agonistic activity and/or with undefined stereochemistry, a total of 103 spiropiperidines analogues with a wide spectrum of activities against the nociceptin/orphanin FQ receptor synthesized by Caldwell JP *et al*. were used as the dataset for molecular modeling in this study [29,30]. *In vitro* biological activities were converted into corresponding p*K*_i_ (−lg*K*_i_) values and used as dependent variables in the QSAR analysis. In approximately a ratio of 4:1, all molecules was divided into training (81 compounds) and test (22) sets. The selection of the test set chemicals obeys the rule that their p*K*_i_ values are randomly but uniformly distributed in the range of the values for the whole set so that the model’s predictive power could be effectively evaluated. Table 2 shows the representative skeletons and activities of the molecules, with all structures and binding affinity values of the dataset listed in supporting information Tables S1–S3.

During the modeling process, the 3D structures of all compounds were subjected to full geometry optimization using the sketch molecule module of Sybyl 6.9 package [50]. Partial atomic charges were calculated by the Gasteiger-Huckel method [51], and energy minimizations were performed by using the Tripos force field [52] and the Powell conjugate gradient algorithm with a convergence criterion of 0.05 kcal/mol.

### 4.2. Conformational Sampling and Alignment

**Figure 6:**
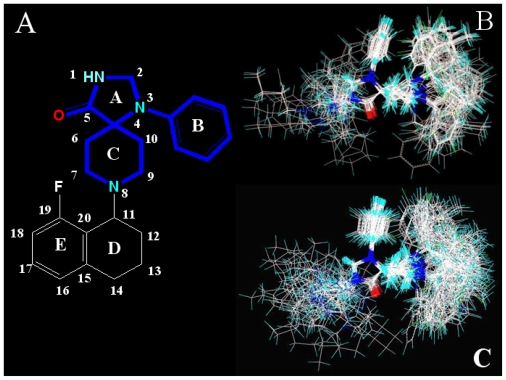
Molecular alignment of compounds in the dataset. (**A**) Common substructure of the molecules is shown in bold based on template compound **32**; (**B**) Ligand-based alignment of all the compounds; (**C**) Receptor-based alignment of all the compounds.

Molecular alignment of compounds is a crucial step in the development of 3D-QSAR models [44]. In order to obtain the best possible 3D-QSAR statistical model, two different alignment rules were adopted. The first rule is the ligand-based alignment. During the process, the most potent molecule (compound **32**) was chosen as a template to fit the remaining training and test sets of compounds by using substructure-alignment function available in Sybyl. The common substructure for the alignment is described in Figure 6A, and the resulting ligand-based alignment model is shown in Figure 6B. The alignment result based on another rule, the receptor-based one, is shown in Figure 6C.

### 4.3. CoMFA and CoMSIA Field Calculation

The CoMFA and CoMSIA models were generated by using Sybyl 6.9 with the default parameters. Detailed algorithms of CoMFA and CoMSIA can be easily referred to many literatures, thus we only introduce the modeling parameters in this work.

To derive the CoMFA and CoMSIA descriptor fields, a 3D cubic lattice with grid spacing of 2 Ǻ in *x*, *y*, and *z* directions, was generated automatically to encompass the aligned molecules. In CoMFA, the steric and electrostatic fields were calculated separately for each molecule using sp^3^ carbon atom probe with a charge of +1.00 and energy cut-off values of 30 kcal/mol for both the steric and electrostatic fields. The probe atom was placed at each lattice point, and its steric and electrostatic interactions with each atom in the molecule were computed using the CoMFA standard scaling.

CoMSIA similarity index descriptors were derived using the same lattice boxes as those used in CoMFA calculations. In CoMSIA, the steric, electrostatic, hydrophobic, and hydrogen-bond (H-bond) donor and acceptor descriptors were calculated using a probe atom of radius 1.0 Ǻ, charge +1.0, and hydrophobicity +1.0. A Gaussian function is used to evaluate the mutual distance between the probe atom and each molecule atom. Because of the different shape of the Gaussian function, CoMSIA similarity indices (*A_F_*) for molecule *j* with atom *i* at grid point *q* are calculated by equation:

(1) $$A_{F,\text{k}}^{q}(j)=-\sum \omega_{probe,k}\;\omega_{ik}\;e^{-\alpha \gamma_{iq}^{2}}$$

where *k* represents the steric, electrostatic, hydrophobic, or hydrogen-bond donor or acceptor descriptor. *ω_probe,k_* is the probe atom with radius 1.0 Ǻ, charge +1, hydrophobicity +1, H-bond donating +1, H-bond accepting +1; *ω_ik_* is the actual value of the physicochemical property *k* of atom *i*; γ*_iq_* is the mutual distance between the probe atom at grid point *q* and atom *i* of the test molecule. The attenuation factor was set to 0.3.

### 4.4. 3D-QSAR Model Generation

In order to generate statistically significant 3D-QSAR models, partial least squares (PLS) regression was adopted to analyze the training set by correlating the variation in their p*K*_i_ values (the dependent variable) with variations in their CoMFA/CoMSIA interaction fields (the independent variables). PLS is a statistical approach that generalizes and combines features from principal component analysis and multiple regressions. When the matrix of predictors has more variables than observations (multicollinearity), PLS is particularly a useful way to predict a set of dependent variables from a large set of independent variables.

Leave-one-out (LOO) cross-validation analysis that one compound was moved away from the dataset and its activity was predicted by the model derived from the rest of the dataset, was performed to evaluate the reliability of the models generated from the PLS analysis. A cross-validated correlation coefficient, *Q*^2^, was subsequently obtained and provided as a statistical index of the predictive power. Then, a non-cross-validation analysis was carried out with the Pearson coefficient (*R*^2^_ncv_) and standard error of estimate (SEE) calculated. Finally, the CoMFA/CoMSIA results were graphically represented by field contour maps, where the coefficients were generated using the field type “Stdev*Coeff”.

In order to evaluate the real predictive ability of the best models generated by the CoMFA/CoMSIA analyses using the training set, the 26 compounds not used in the model generation are used as the external validation set. A predictive R value was then obtained with the following formula:

(2) $$R_{pred}=\sqrt{\frac{SD-PRESS}{SD}}$$

where SD denotes the sum of squared deviation between the biological activities of the test set molecule and the mean activity of the training set molecules; PRESS represents the sum of squared deviations between the experimental and predicted activities of the test molecules, respectively.

### 4.5. Molecular Docking

Molecular docking was carried out by the Surflex-dock module (V 2.51) [53] of an advanced version of Sybyl-X 1.1 [54] to understand the detailed binding model for the active site of NOP receptor with its ligands. In Surflex-docking, protomol was a computational representation of the intended binding site to which putative ligands were aligned and its construction was based on the protein residues proximal to the native ligand and on parameter settings to produce a small and buried docking target. Up to now, the protein structure has not been resoluted and the identities of our homology modeling models are below 30%. However, Luo HB from School of Pharmaceutical Sciences, Sun Yat-Sen University, who has done the similar studies on NOP receptor and used homology modeling to make a protein, is very kind to provide us his protein structure for our study. The molecular docking process is summarized as the following steps: First, the template protein structure was imported into Surflex. Then the protomol was generated using a ligand approach. Two parameters, protomol_bloat and protomol_threshold, which respectively determine how far from a potential ligand the site should extend and how deep into the protein the atomic probes used to define the protomol can penetrate, are set at 0 and 0.60 respectively. Finally, each conformer of all 103 agonists was docked into the binding site 20 times. The Hammerhead scoring function [55] is used to score the molecules in the putative poses. During the present molecular docking process, the protein was considered to be rigid, and the ligand molecules were flexible. All other parameters were setting at default values.

### 4.6. Molecular Dynamics Simulations

After docking analysis, the docked structure of compound **32** was applied in the MD simulations using the Amber 10 [56]. The general atom force field (GAFF) [57] and the standard AMBER force field for bioorganic systems (ff03) [58] were used to model the ligand and protein respectively. The docked structure was neutralized with 9 counter chloridion ions and solvated in a rectangular box of TIP3P [59] water, which kept a minimum distance of 12 Å between the solute and each face of the box (74.984 × 97.951 × 67.771 Å^3^). The total number of the atoms of the simulation system was 40091 including the complex and waters. The cutoff distance was kept to 10 Å to compute the non-bonded interactions. All simulations were performed under periodic boundary conditions. To remove possible bad contacts, the complex was energy minimized by a multistep procedure including 9500 conjugate-gradient steps followed by 500 steepest-descent steps. Constant volume dynamics with a cutoff of 10 Å was chosen. SHAKE [60] was turned on for bonds involving H-atoms.

In the simulation process, first, the minimized system was heat up to 300 K at a constant rate of 6 K/ps while the protein atoms were constrained. The second step depended on a 50 ps pressure-constant period to raise the density and keep the complex atoms constrained. The third step was a 500 ps Langevin dynamics calculation with a collision frequency of 1 ps^−1^, which was performed with a 2 fs time step at a constant temperature of 300 K. Finally, the production phase was run for 5 ns with a 2 fs time step. The long-range electrostatics was treated by using the particle-mesh-Ewald (PME) method [61] with default values.

## 5. Conclusion

In this paper, the ligand- and receptor-based 3D-QSAR studies of 107 spiropiperidines analogues as agonists of nociceptin/orphanin FQ receptor have been performed using CoMFA and CoMSIA tools. From the resultant model, the high *Q*^2^, *R*^2^_ncv_, and *R*^2^_pred_ values prove that the 3D-QSAR models developed in this work are statistically reliable and predictable. The resulting contour maps produced by the best CoMSIA model provide useful information about the intermolecular interactions of agonists with the surrounding environment. The good consistency between the 3D-QSAR, the docking and MD modeling results, once again, demonstrates the reliability of the model. The newly obtained 3D model of NOP may serve as a basis for development of novel agonists with enhanced affinity. Overall, the conclusions are summarized as follows (with compound 32 as a reference):

1. Substituents with bulky, electro-sensitive, hydrophilic, H-bond donor at position-1, bulky hydrophilic substituents at positions-11 and -12 and minor hydrophobic substituents at positions-6, -7 and -9 of ring C may be helpful to enhance potency. Electronegative H-bond acceptor at position-5, bulky hydrophobic substituents at position-19, bulky substituents at position-20, electropositive substituents at positions-4 and -11, electronegative substituents at position-8 and hydrophilic substituents at position-17 and ring B can all enhance the activity.
2. The binding site of N-substituted spiropiperidine-based NOP agonists is mostly a large hydrophobic pocket formed by Trp116, Trp211, Trp276, Val126, Phe220, Phe106, Ile127 and Pro207 residues. The H-bonds formed by polar residues Thr103 and Tyr309 can be identified as potential factors greatly impacting the binding affinity of compound **32**.

## Supplementary Material



## Figures and Tables

**Table 1:** Summary of CoMFA and CoMSIA results.

PLS Statistics	Ligand-Based Model		Receptor-Based Model	

	CoMFA	CoMSIA	CoMFA	CoMSIA
*Q^2^*	0.229	0.503	0.047	0.111
*R^2^*_ncv_	0.621	0.921	0.196	0.527
SEE	0.507	0.237	0.728	0.559
*F*	41.472	120.623	19.064	86.880
*R^2^*_pred_	0.712	0.788	0.227	0.385
SEP	0.723	0.596	0.793	0.766
PLS components	3	7	1	1
**Contribution**				
Steric	0.528	0.122	0.435	0.172
Electrostatic	0.472	0.355	0.565	0.242
Hydrophobic		0.258		0.249
H-bond donor		0.266		0.337

*Q*^2^: Leave-one-out cross-validated correlation coefficient; *R*^2^_ncv_: non-cross-validated correlation coefficient; SEE: standard error of estimate; *F*: ratio of *R*^2^_ncv_ explained to unexplained *R*^2^_ncv_/(1 − *R*^2^_ncv_); *R*^2^_pred_: predicted correlation coefficient for the test set of compounds; SEP: standard error of prediction; PLS components: the optimal number of principal components.

**Table 2:** Representative skeleton, molecular structure and binding affinity (*K*_i_, nM) of spiropiperidine analogues.

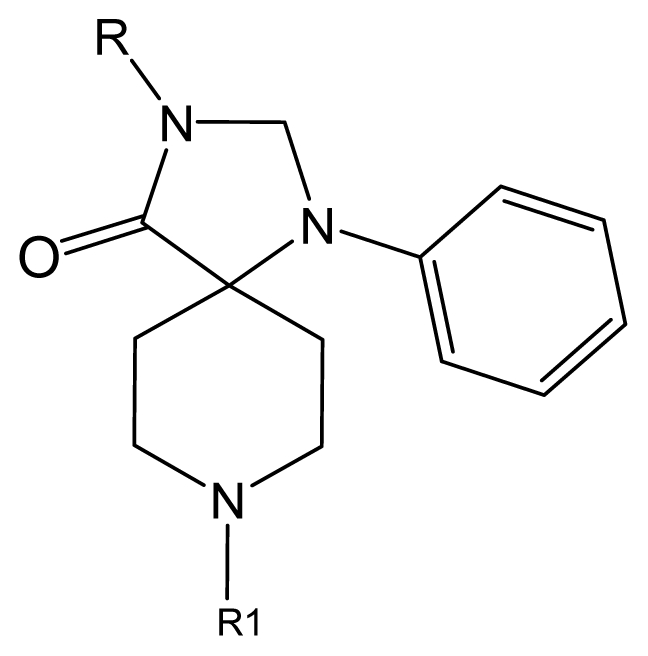							
No.	R	R_1_	K_i_	No.	R	R_1_	K_i_
5	H	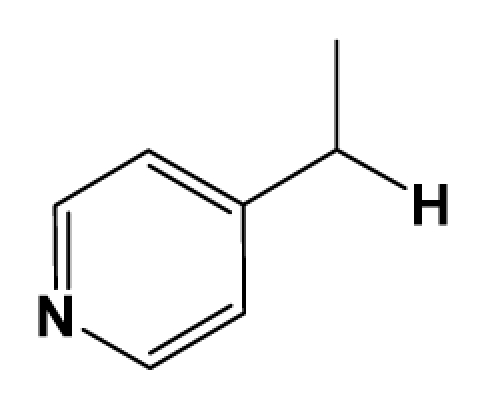	824.0	44 #	Bu	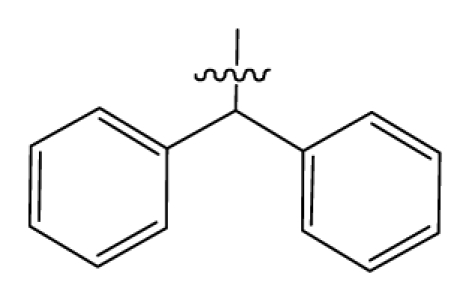	57
6	H	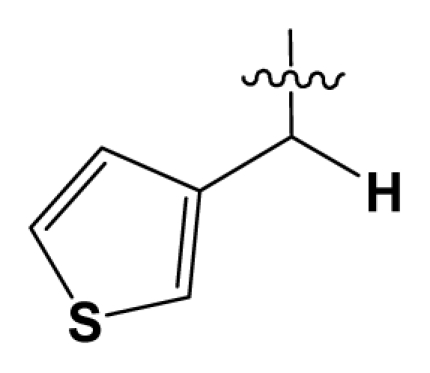	14.0	47	*c*-BuCH_2_-	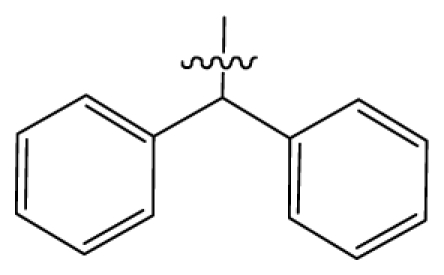	53
11	H	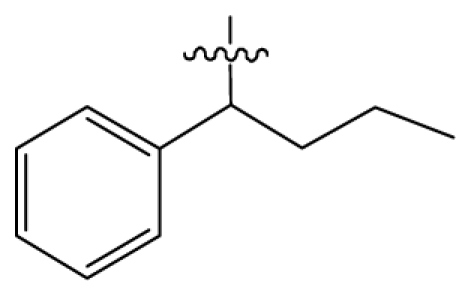	2.4	54 #	HO(CH_2_)_2_-	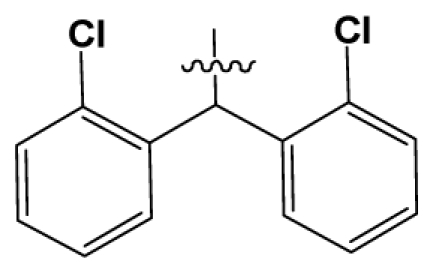	18.5
16	H	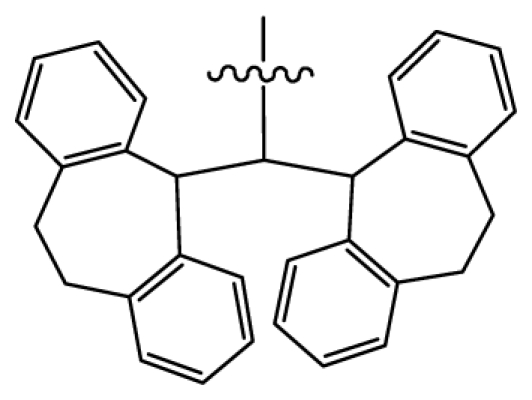	225.0	69	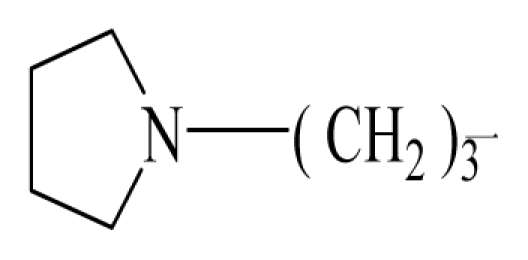	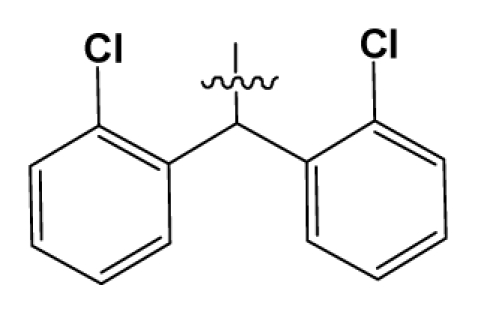	3.2
19	H	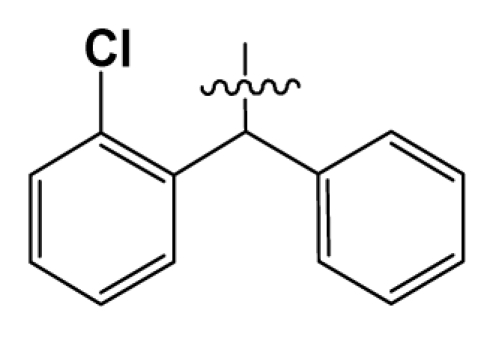	9.0	75	*c*-BuNH(CH_2_)_2_-	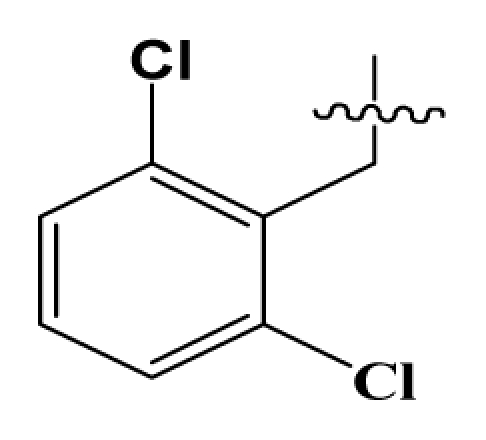	0.5
26	H	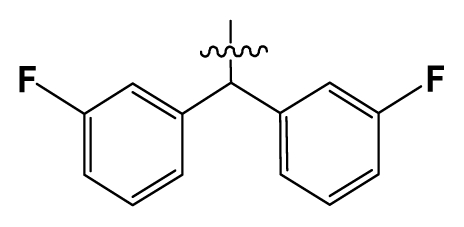	250.0	79	Et_2_N(CH_2_)_2_-	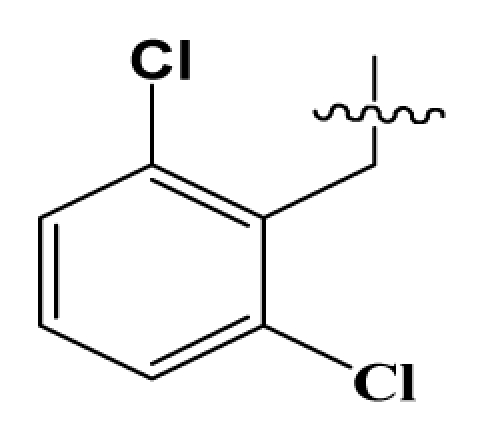	1.0
30	H	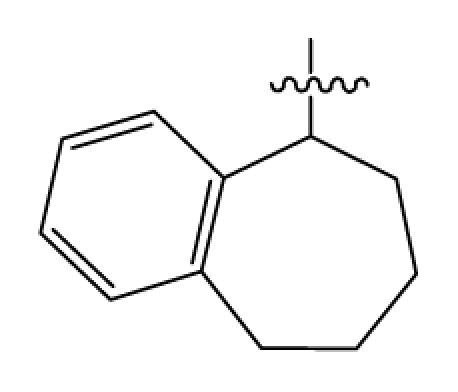	14.5	87	*c*-Pentyl NH(CH_2_)_2_-	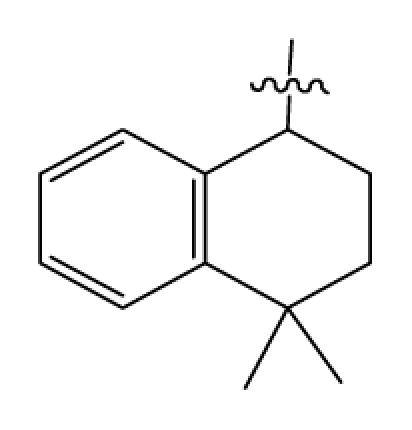	0.9
32	H	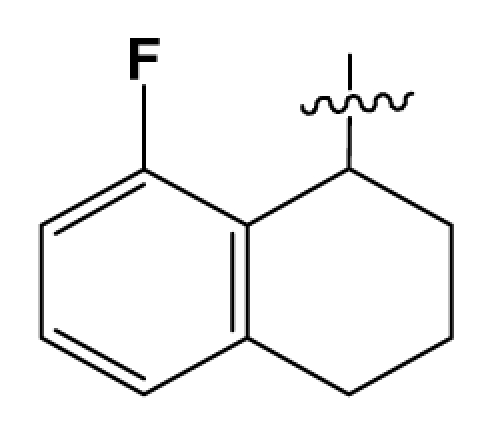	0.3	90 #	CH_2_=CHCH_2_NH(CH_2_)_2_-	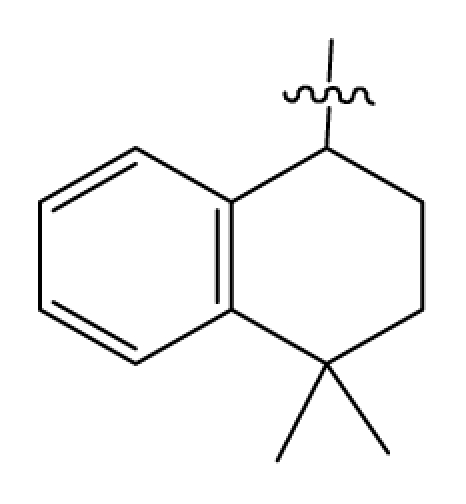	0.9
36 #	H	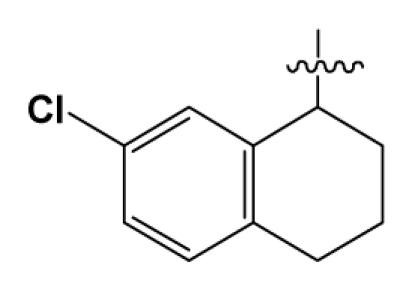	10.8	97	*i*-AmylNH(CH_2_)_2_-	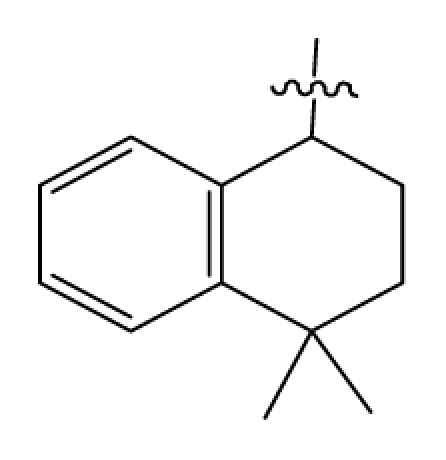	0.4
39 #	H	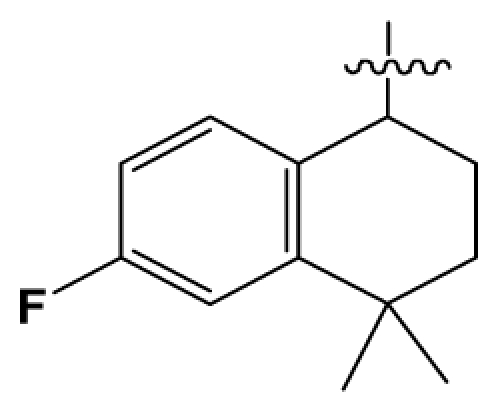	8.4	103	BuNH(CH_2_)_2_-	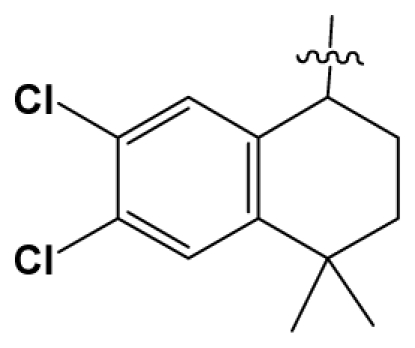	12.2

# Molecules belonging to the test set.
