# Supplementary material for: Structural Requirements of N-Substituted Spiropiperidine Analogues as Agonists of Nociceptin/Orphanin FQ Receptor
Source: Int J Mol Sci. 2011 Dec 6;12(12):8961–81. doi: 10.3390/ijms12128961 (PMC3257111; doi:10.3390/ijms12128961)
Supplement: Supplementary file 1 [file ijms-12-08961-s001.pdf]

Table S1. Structures of compounds with skeleton type A in the data set.

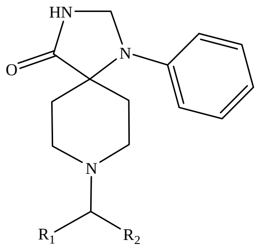

Skeleton type A

Compound	R_1	R_2	K_i (nM)
1	Ph	H	11.0
2	Cyclohexyl	H	61.2
3	2-Pyridyl	H	119.0
4	3-Pyridyl	H	133.0
5	4-Pyridyl	H	824.0
6	3-Thienyl	H	14.0
7	2-Cl-Ph	H	3.2
8	2,6-Cl_2-Ph	H	2.3
9	Ph	Me	3.5
10	Ph	Et	17.0
11	Ph	Propyl	2.4
12	Ph	Butyl	1.1
13	Ph	Isoamyl	1.5
14	Ph	Cyclopentyl	4.6
15	Ph	Ph	23.0
16	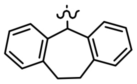	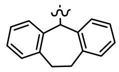	225.0
17	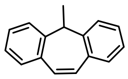	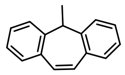	36.5
18	2-F-Ph	Ph	10.8
19	2-Cl-Ph	Ph	9.0
20	2-Me-Ph	Ph	11.5
21	4-Cl-Ph	Ph	140.0
22 [#]	2-Me-Ph	2-Me-Ph	9.0
23	2-Cl-Ph	2-Cl-Ph	6.8
24	3-Cl-Ph	3-Cl-Ph	249.0
25	2-F-Ph	2-F-Ph	6.3
26 [#]	3-F-Ph	3-F-Ph	250.0
27	4-F-Ph	4-F-Ph	49.0

[#] Molecules belonging to the test set.

Table S2. Structures of compounds with skeleton type B in the data set.

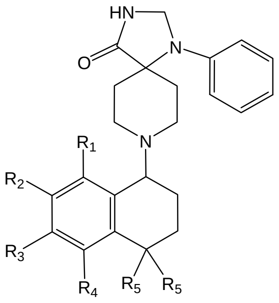

Skeleton type B

Compound	R_1	R_2	R_3	R_4	R_5	n	K_i (nM)
28 #	H	H	H	H	H	0	1.2
29	H	H	H	H	H	1	1.4
30	H	H	H	H	H	2	14.5
31	H	H	H	H	CH_3	1	1.3
32	F	H	H	H	H	1	0.3
33	H	F	H	H	H	1	0.9
34	H	H	H	F	H	1	0.8
35 #	Cl	H	H	H	H	1	2.6
36 #	H	Cl	H	H	H	1	10.8
37 #	H	H	H	Cl	H	1	2.0
38	H	H	Cl	H	CH_3	1	1.4
39 #	H	H	F	H	CH_3	1	8.4
40	H	Cl	Cl	H	CH_3	1	15.1

Molecules belonging to the test set.

Table S3. Structures of compounds with skeleton type C in the data set.

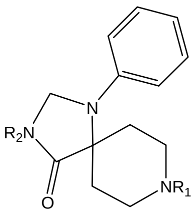

Skeleton type C

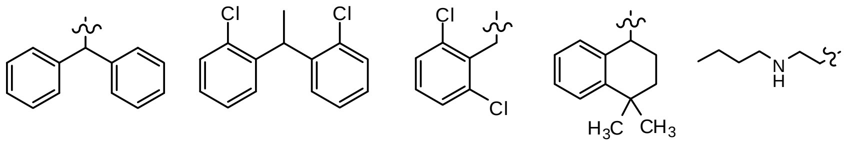

A B C D E

Compound	R_1	R_2	K_i (nM)
41	A	Me	31
42	A	Et	84
43	A	Pr	34
44 #	A	Bu	57
45	A	*i*-Pr	66
46	A	*c*-$PrCH_2$-	83
47	A	*c*-$BuCH_2$-	53
48 #	A	*c*-$HexylCH_2$-	89
49	A	Propargyl	234
50 #	A	Allyl	203
51	B	Bu-	48.7
52	B	*i*-Amyl	56
53	B	$CH_3OC(O)CH_2$-	19.5
54 #	B	$HO(CH_2)_2$-	18.5
55	B	$MeO(CH_2)_2$-	26
56 #	B	$NH_2(CH_2)_2$-	47.8
57 #	B	$CH_3NH(CH_2)_2$-	4.05
58	B	$EtNH(CH_2)_2$-	2.1
59	B	*i*-$PrNH(CH_2)_2$-	2.55
60	B	*c*-$PentylNH(CH_2)_2$-	8.65
61	B	*c*-$HexylNH(CH_2)_2$-	5
62 #	B	$(CH_3)_2N(CH_2)_2$-	3.5
63	B	*c*-$PrNH(CH_2)_2$-	3.7
64	B	$(i\text{-}Pr)_2N(CH_2)_2$-	12.1
65 #	B	$BuNH(CH_2)_2$-	2.15
66 #	B	*i*-$BuNH(CH_2)_2$-	2.75
67	B	*c*-$HexylCH_2NH(CH_2)_2$-	3.8
68	B	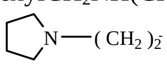	2.25
69	B	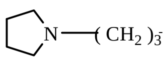	3.2
70	B	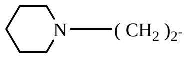	8
71	C	$CH_3NH(CH_2)_2$-	0.8
72 #	C	$EtNH(CH_2)_2$-	0.7
73	C	*i*-$PrNH(CH_2)_2$-	0.7
74	C	*c*-$PrCH_2NH(CH_2)_2$-	0.5
75 #	C	*c*-$BuNH(CH_2)_2$-	0.5
76	C	$PrNH(CH_2)_2$-	0.6
77	C	*i*-$BuNH(CH_2)_2$-	0.5
78 #	C	$BuNH(CH_2)_2$-	0.4

Table S3. *Cont.*

Compound	R_1	R_2	K_i (nM)
79	C	$Et_2N(CH_2)_2$-	1.0
80	C	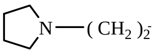	2.3
81 #	D	Pr–	5.4
82	D	$CH_3C(O)CH_2$-	4.5
83	D	$HO(CH_2)_2$-	1.7
84	D	$CH_3NH(CH_2)_2$-	2.1
85	D	$EtNH(CH_2)_2$-	1.6
86 #	D	*i*-$PrNH(CH_2)_2$-	1.4
87	D	*c*-$PentylNH(CH_2)_2$-	0.9
88	D	*c*-$HexylNH(CH_2)_2$-	0.9
89	D	$PrNH(CH_2)_2$-	1.0
90 #	D	CH_2=$CHCH_2NH(CH_2)_2$-	0.9
91	D	*c*-$BuNH(CH_2)_2$-	1.5
92 #	D	*c*-$PrCH_2NH(CH_2)_2$-	0.8
93	D	*i*-$BuNH(CH_2)_2$-	0.5
94	D	(*i*-$Pr)_2NH(CH_2)_2$-	6.7
95	D	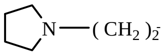	1.4
96	D	$BuNH(CH_2)_2$-	0.5
97	D	*i*-AmylNH(CH2)$_2$-	0.4
98	D	*c*-$HexylCH_2NH(CH_2)_2$-	0.7
99	D	$BnNH(CH_2)_2$-	3.6
100	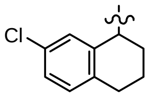	E	15.9
101	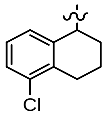	E	1.8
102	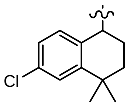	E	0.6
103	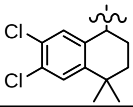	E	12.2

Molecules belonging to the test set.

© 2011 by the authors; licensee MDPI, Basel, Switzerland. This article is an open access article distributed under the terms and conditions of the Creative Commons Attribution license (http://creativecommons.org/licenses/by/3.0/).
